# Marker Density and Models to Improve the Accuracy of Genomic Selection for Growth and Slaughter Traits in Meat Rabbits

**DOI:** 10.3390/genes15040454

**Published:** 2024-04-03

**Authors:** Wenjie Li, Wenqiang Li, Zichen Song, Zihao Gao, Kerui Xie, Yubing Wang, Bo Wang, Jiaqing Hu, Qin Zhang, Chao Ning, Dan Wang, Xinzhong Fan

**Affiliations:** 1 Department of Animal Genetics and Breeding, Shandong Agricultural University, Taian 271000, China; wjli@ahau.edu.cn (W.L.); 2021010079@sdau.edu.cn (W.L.); zichen1006@outlook.com (Z.S.); xiekerui666@163.com (K.X.); wangbo8604@126.com (B.W.); jqh0609@sdau.edu.cn (J.H.); qzhang@sdau.edu.cn (Q.Z.); ningchao@sdau.edu.cn (C.N.); 2 Department of Animal Genetics and Breeding, University of Anhui Agricultural, Hefei 230031, China; 3 Key Laboratory of Efficient Utilization of Non-Grain Feed Resources (Co-Construction by Ministry and Province), College of Animal Science and Veterinary Medicine, Shandong Agricultural University, Ministry of Agriculture and Rural Affairs, Taian 271000, China

**Keywords:** genomic selection, marker density, GBLUP models, low-coverage whole-genome sequencing, meat rabbits

## Abstract

The selection and breeding of good meat rabbit breeds are fundamental to their industrial development, and genomic selection (GS) can employ genomic information to make up for the shortcomings of traditional phenotype-based breeding methods. For the practical implementation of GS in meat rabbit breeding, it is necessary to assess different marker densities and GS models. Here, we obtained low-coverage whole-genome sequencing (lcWGS) data from 1515 meat rabbits (including parent herd and half-sibling offspring). The specific objectives were (1) to derive a baseline for heritability estimates and genomic predictions based on randomly selected marker densities and (2) to assess the accuracy of genomic predictions for single- and multiple-trait linear mixed models. We found that a marker density of 50 K can be used as a baseline for heritability estimation and genomic prediction. For GS, the multi-trait genomic best linear unbiased prediction (GBLUP) model results in more accurate predictions for virtually all traits compared to the single-trait model, with improvements greater than 15% for all of them, which may be attributed to the use of information on genetically related traits. In addition, we discovered a positive correlation between the performance of the multi-trait GBLUP and the genetic correlation between the traits. We anticipate that this approach will provide solutions for GS, as well as optimize breeding programs, in meat rabbits.

## 1. Introduction

Meat rabbits are ideal meat-producing herbivorous animals with high feed conversion ratios (43–50% for high-grain diets and 26–33% for high-forage diets) [1]. Rabbit meat is considered a very nutritious and healthy food due to its low levels of fat, cholesterol, and sodium, as well as its rich protein content [2]. To accelerate the development of the meat rabbit industry, it is necessary to breed for important economic traits, such as growth traits, slaughter traits, and feed conversion ratios, which are falling behind those of other livestock.

Meat rabbits, as a niche livestock breed, are relatively backward in breeding, and the best linear unbiased prediction (BLUP) method is most commonly used. With the mastery of the economic traits of most meat rabbit breeds (strains), utilizing hybrid advantages to improve the production of meat rabbits has also become an effective way to improve important economic traits [3]. However, these methods are generally influenced by environmental, genetic, and environment–gene interaction factors. Genomic selection (GS) was put forward by Meuwissen et al. in 2001 [4]. Since then, various GS studies have sought to improve the accuracy of genomic-estimated breeding values (GEBVs). GS has been found to effectively increase the rates of genetic gain in Australian cattle herds [5]. At the same time, multi-trait GS model analyses are better than their single-trait counterparts in selecting for multiple related traits [6]. The motivations for utilizing GS include shortening the generation intervals, and enhancing the power and accuracy of selection, contributing to genetic improvement [7,8]. It has been found that GS greatly improves the selection accuracy for the early and late trait in laying hens [9], while modeling and empirical investigations on GS have achieved improvements in the production of many animals [10,11,12]. GS has been effectively implemented in animal breeding programs for over two decades, and it has been extensively utilized in the selection of virtually all significant livestock species to date [12,13,14,15,16]. However, the lack of inexpensive SNP microarrays and the high cost of genotyping through resequencing have delayed the application of GS for rabbits [17].

The accurate detection of variants usually requires sequencing at the high depth of 10× [18]. However, this remains too expensive for large-scale GS applications, though the sequencing price per gigabase is decreasing. A well-established approach is to perform lcWGS at a depth of about 1× or less, combined with genotypic imputation, to obtain the variants across the whole genome. This method has been utilized in variant genotyping for genome-wide association studies and GS for humans, animals, and plants [19,20,21,22,23,24].

Various factors, including marker densities, heritability, models, and the interaction between them, appear to affect the prediction accuracy in GS [25,26]. One of the most researched elements, marker density, significantly affects the accuracy of GS prediction. High-density markers have been suggested to improve predictive accuracy [27,28], and it is agreed that a higher number of markers typically produces higher accuracy up to a plateau [29,30]. For genome resequencing, the marker density most suitable for GS in meat rabbits, i.e., the density at which the plateau is reached, remains unknown and needs to be explored further, as the GS model’s dimensionality may decrease depending on the number of valid SNPs.

The numerous studies on GS have mostly been limited to single traits for many years, although they have not been very predictive [31]. In recent years, multiple-trait-based GS approaches have been explored, which could provide more accurate GEBVs by exploiting correlated structural information between traits [32]. In the practice of breeding livestock, there are often strong genetic correlations between multiple growth/slaughter traits; thus, a multi-trait model might improve the evaluation of GEBVs [32,33]. There have been few GS studies for meat rabbits, and the accuracy of GEBVs evaluated using single-trait or multi-trait models is unclear.

In this study, we used imputed genotype data to estimate the optimal marker density for heritability estimation and GS for growth and slaughter traits in meat rabbits and compared the GS performance of single-trait and multiple-trait genomic best linear un-biased prediction (GBLUP) models for these traits. This study aimed to provide a strategy for the next step in the molecular breeding of meat rabbits and to optimize their breeding programs.

## 2. Materials and Methods

### 2.1. Animal Phenotypes and Genotypes

All the experimental procedures in the present study were approved by the College of Animal Science and Technology, Shandong Agricultural University (SDAUA). The animal ethics committee of SDAUA gave ethical approval for the animal work (No. SDAUA-2020-110, 10 September 2019). The 1515 Kanda V line meat rabbits used in this study were from Kangda Breeding Farm (Qingdao, Shandong, China), comprising 5 batches and 399 litters, of which 1159 (516 males and 643 females) were documented for growth traits and 798 (333 males and 465 females) were slaughtered. All the rabbits were from five batches born in 2019–2021 and were kept in standardized rabbit hutches under uniform conditions regarding factors including diet, water, and temperature. The growth traits, including the weights at 35 (W35), 49 (W49), and 70 days (W70) of age, were recorded, and the feed conversion ratio (FCR) from 49 to 70 days was calculated using weight gain/feed intake. At 70 days of age, the rabbits were slaughtered, and the slaughter traits, including the eviscerated weight (EW), half-eviscerated weight (HEW), kidney weight (KW), net head weight (NHW), hind leg weight (HLW), and fore leg weight (FLW), were determined.

Blood samples were collected from the slaughtered rabbits, while ear tissue samples were collected from others. Genomic DNA was extracted using the Qiagen MinElute kit. Paired-end (PE150) libraries with an insert size of ~350 bp were sequenced on the NovaSeq 6000 or DNBSEQ-T7 platform. The samples were sequenced with an average genomic coverage of 1.74×, with the read depth varying from 0.80× to 16.41× (Supplementary Table S1). We first processed the resequencing data, and the read quality was evaluated using FastQC (https://www.bioinformatics.babraham.ac.uk/projects/fastqc/) accessed on 20 May 2021. Raw data connectors and low-quality reads were removed using Trimmomatic [34]. After the initial QC, the data were compared to the reference genome using Bwa [35] to determine the location of each read on the reference genome. Then, the genotypes were imputed using STITCH [36] and Beagle v5.1 [37] according to Wang et al. [38]. A total of 20,125,019 high-quality SNPs (imputation accuracy above 98%) were used after stringent quality control.

### 2.2. Models

The genomic best linear unbiased prediction method [35] was used to obtain GEBVs under single-trait or multiple-trait GBLUP-based models.

The univariate linear mixed models (uvLMMs) are as follows:

y=Xb+Za+e

where y is the vector of phenotypes, b is the vector of fixed effects (including sex, batch, environmental effect, and year of birth), X is the incidence matrix for b, a is the vector of random genetic effects, Z is the incidence matrix for a, e is a vector of random residuals, $Var\left(a\right)=G\sigma_{a}^{2}$, $\sigma_{a}^{2}$ is the additive genetic variance, and G is the genomic relationship matrix. The distributions of random effects are

$$a\sim N(0,\;G\sigma_{a}^{2}),\;e\sim N(0,\;I\sigma_{e}^{2})$$

We designed different combinations of growth and slaughter traits to estimate GEBVs using multivariate linear mixed models (mvLMMs). As an example, the mvLMM for the two-trait model is as follows:

y=Xb+Za+e

where y is the vector of individual phenotypic values, y $=\left[\begin{array}{c} y_{1}\\ y_{2} \end{array}\right]$; b is the vector of fixed effects (including sex, batch, environmental effect, and year of birth), $b=\left[\begin{array}{c} b_{1}\\ b_{2} \end{array}\right]$; X is the correlation matrix of individual fixed effect coefficients, X $=\left[\begin{array}{c} X_{1}\;0\\ 0\;X_{2} \end{array}\right]$; a is the additive genetic effects, $a=\left[\begin{array}{c} a_{1}\\ a_{2} \end{array}\right]$; Z is the a correlation matrix, Z $=\left[\begin{array}{c} Z_{1}\;0\\ 0\;Z_{2} \end{array}\right]$; and e is the random residual vector, $e=\left[\begin{array}{c} e_{1}\\ e_{2} \end{array}\right]$.

Assume $\left[\begin{array}{c} a_{1}\\ a_{2} \end{array}\right]\sim N(0,\;M⨂G),\;M=\left[\begin{array}{c} \sigma_{a_{1}}^{2}\;\sigma_{a_{12}}\\ \sigma_{a_{12}}\;\sigma_{a_{2}}^{2} \end{array}\right]$ is the variance–covariance matrix of the estimated breeding values for the two-trait genomes, and G is the matrix of the genetic relationships constructed from the information of the genomic markers in the GBLUP method for the single traits. Assume $\left[\begin{array}{c} e_{1}\\ e_{2} \end{array}\right]\sim N(0,\;R⨂I$, $R=\left[\begin{array}{c} \sigma_{e_{1}}^{2}\;\sigma_{e_{12}}\\ \sigma_{e_{12}}\;\sigma_{e_{2}}^{2} \end{array}\right]$ is the variance–covariance matrix of the residuals of the two traits. Here, $\sigma_{a_{1}}^{2}$ represents the variance of the additive effect of trait 1, $\sigma_{a_{2}}^{2}$ represents the variance of the additive effect of trait 2, $\sigma_{a_{12}}$ represents the additive effect covariance of traits 1 and 2, $\sigma_{e_{1}}^{2}$ represents the variance of the random residual effect of trait 1, $\sigma_{e_{2}}^{2}$ represents the variance of the random residual effect of trait 2, and $\sigma_{e_{12}}$ represents the random residual effect covariance of traits 1 and 2.

In this study, the VanRaden [39] method was used to construct the G matrix using the completed imputed genotype data via GMAT [40]. The applied models, as well as the variance and covariance components in the estimated breeding values of the individual genomes, were obtained via the DMU package (https://dmu.ghpc.au.dk/dmu/) accessed on 21 August 2021) [41].

### 2.3. Marker Densities

To assess the effect of marker density on the heritability estimation and genomic prediction, we randomly selected 0.5 K, 1 K, 3 K, 5 K, 10 K, 50 K, 100 K, 500 K, 1 M, and 2 M markers. Then, we utilized the randomly selected markers to construct a genomic relationship matrix while estimating the heritability and genomic breeding values using univariate linear mixed models. To obtain stable results, we ran the extraction process 30 times for each marker density.

### 2.4. Cross-Validation

We used 10-fold cross-validation (CV) to assess the genomic prediction accuracy by randomly dividing the dataset into 10 subsets of roughly equal numbers. For each cross-validation, nine subsets were selected and we considered the training population (reference population) to estimate the genetic parameters and genomic breeding values. For the remainder subset, we considered the validation population (candidate population) to verify the accuracy, and this was performed for 10 cycles to complete one validation. The accuracy of the estimated genomic breeding values in this study was determined using the coefficient of the correlation between the estimated breeding values and the phenotypic values. The formula is expressed as follows:

$$r=\frac{\mathrm{cov}(a,p)}{\sqrt{\mathrm{var}(a)\mathrm{var}(p)}}$$

where var(a) represents the variance of the estimated breeding values, var(p) represents the variance of the phenotypic values, and cov(a, p) represents the covariance of the estimated breeding values with the phenotypic values.

In this study, we used a tow-sample *t*-test to determine whether the predictive accuracy of the two experiments (varied marker densities or models) were significantly different from each other.

## 3. Results

### 3.1. The 50 K Markers as a Baseline for Estimating Heritability for Meat Rabbits

**Figure 1:**
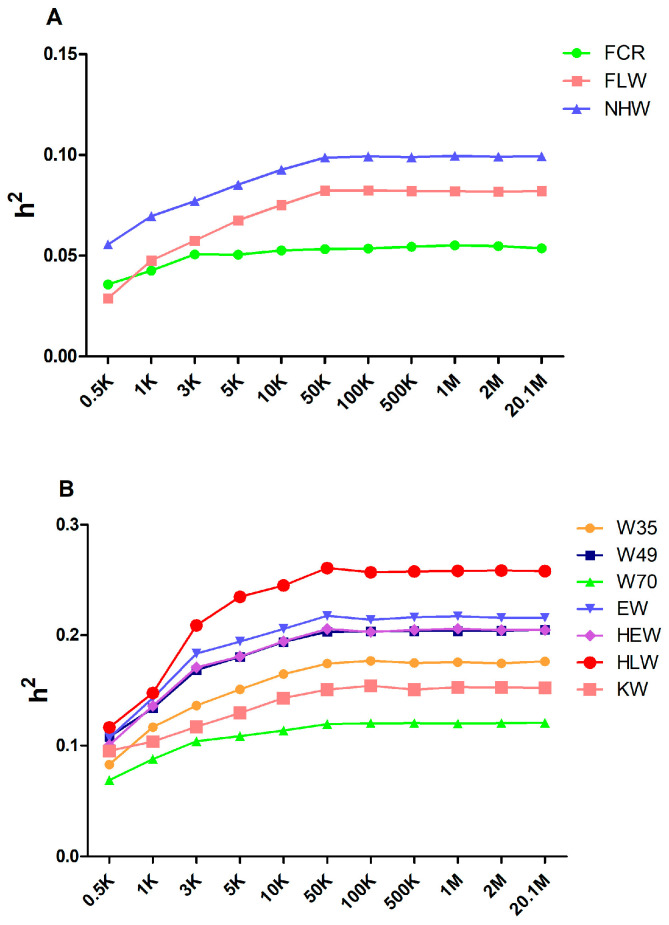
The estimation of the heritability of growth and slaughter traits with various marker densities. The changing estimated heritability for low (**A**) and medium (**B**) heritability traits. Traits were classified into two categories based on the heritability estimated from the original sequencing data: low heritability (<0.1) and medium heritability (0.1–0.3).

To generate varied marker densities, we randomly selected 0.5 K, 1 K, 3 K, 5 K, 10 K, 50 K, 100 K, 500 K, 1 M, and 2 M from the original sequencing markers and repeated the extraction process 30 times for each marker density to decrease the sampling error. We grouped all the traits into two categories based on the estimated heritability of the original sequenced markers, i.e., low heritability (<0.1) and medium heritability (0.1–0.3). Figure 1A,B display the average estimated heritability of 30 randomly selected markers for each marker density. We found that, when the marker density increased, the estimated heritability increased quickly between 0.5 K and 50 K. The estimated heritability maintained an increase until reaching a plateau phase, while the marker density continued to increase after 50 K. The heritability estimated from 20.1 M genomic markers is shown in Table 1.

### 3.2. The 50 K Markers Achieve the Required Prediction Accuracy for the Meat Rabbits

**Figure 2:**
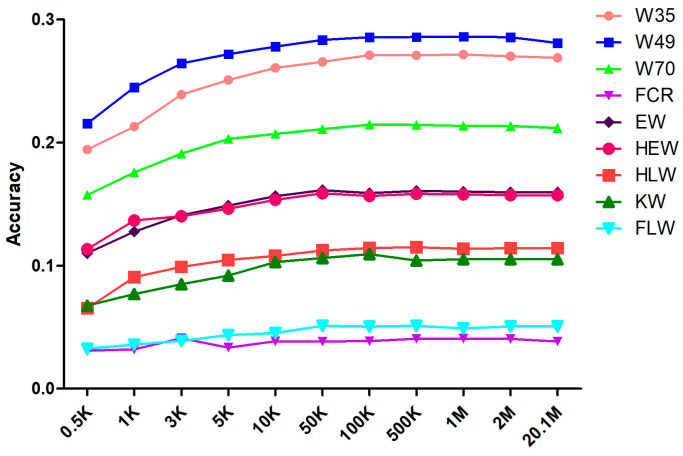
Cross-validation of mean prediction accuracy under different marker densities.

The prediction accuracy for each attribute was calculated by averaging the results of 30 randomly selected cross-validations (Table 2). The change in prediction accuracy with increasing marker density is shown in Figure 2. For each trait, the average prediction accuracy, regardless of marker density, was below 0.3. We discovered that, as the marker density was increased from 0.5 K to 50 K, the prediction accuracy increased quickly, following the trend of estimated heritability. At a marker density of 50 K, the prediction accuracy was essentially flat compared to that of the original sequenced marker results, whereas the prediction accuracy barely fluctuated when the marker density increased. Supplementary Table S2 lists the significance of the differences in prediction accuracy at different marker densities. At marker densities of 50 K and 100 K, the differences in accuracy were not significant for any of the traits, whereas at marker densities of 50 K and 10 K, the differences in accuracy were significant for the traits W49, W70, and FLW.

### 3.3. Accuracy of the Genomic Prediction

**Figure 3:**
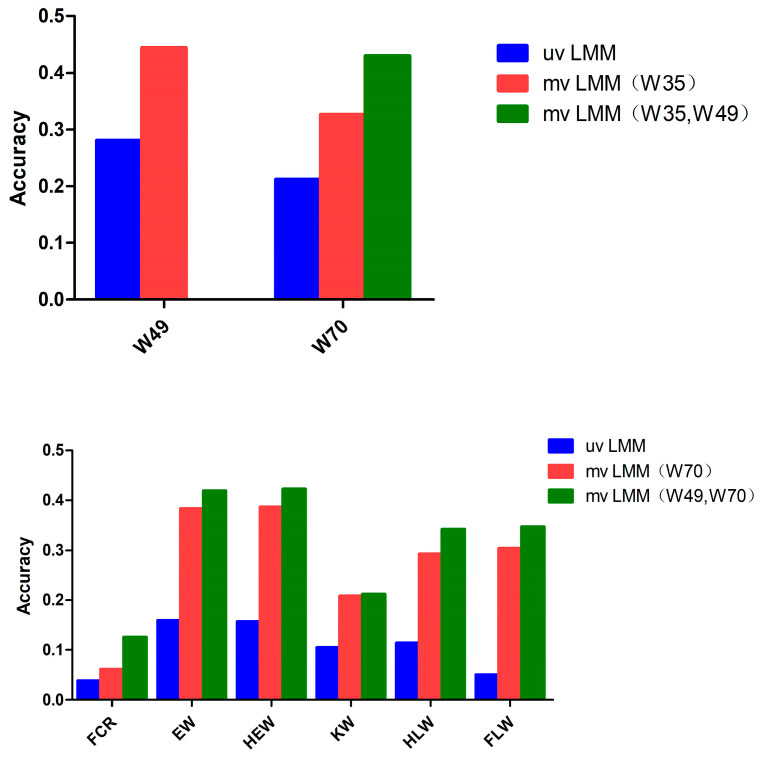
Cross-validation of different trait combination models for GEBVs. uvLMM: a univariate linear mixed model; mvLMM (W35): a multivariate linear mixed model based on the 35-day weight trait; mvLMM (W70): a multivariate linear mixed model based on the 70-day weight trait; mvLMM (W35, W49): a multivariate linear mixed model based on the 35-day and 49-day weight traits; mvLMM (W49, W70): a multivariate linear mixed model based on the 49-day and 70-day weight traits.

GEBV data were analyzed by using the multi-trait models with different combinations of traits. The accuracy of the two-trait model in estimating breeding values could be improved for almost all traits compared to single traits (0.444 vs. 0.281 for W49, 0.326 vs. 0.212 for W70, 0.044 vs. 0.038 for FCR, 0.383 vs. 0.160 for EW, 0.386 vs. 0.157 for HEW, 0.208 vs. 0.105 for KW, 0.293 vs. 0.114 for HLW, and 0.304 vs. 0.051 for FLW; see Figure 3). In terms of genomic prediction, for W49, the prediction accuracy when using the multiple-trait model increased by 58% compared to when using the single-trait model; for W70, the prediction accuracy when using the multiple-trait model increased by 103% compared to when using the single-trait model; for FCR, EW, HEW, KW, HLW, and FLW, the prediction accuracy when using the multiple-trait model increased significantly compared to when using the single-trait model. In addition, as shown by Table 3 and Table 4, the improvement in the accuracy of the breeding values estimated through the multi-trait model was almost proportional to the genetic correlation between the traits.

Cross-validation experiments showed that the multi-trait model could further improve the prediction accuracy if the observed traits with a closer genetic correlation were selected; as the number of early observed traits increased, the prediction accuracy among different traits within a certain range almost always improved (Figure 3; Table 3).

## 4. Discussion

Genomic selection improves the efficiency and accuracy of breeding and has potential research and economic value for improving animal performance, promising to accelerate genetic gain [8,42,43]. Low-coverage whole-genome sequencing (LcWGS) coupled with genotype imputation technology offers significant cost savings compared to whole-genome high-density genotyping [20,24], especially for species without SNP arrays (e.g., rabbits) [44]. In order to improve the breeding process, it was necessary to assess various marker densities and the effectiveness of the GS model in order to implement GS in the breeding of meat rabbits.

### 4.1. Appropriate Marker Density for Heritability Estimation

Traditionally, pedigree information was commonly used to estimate the heritability of various traits in rabbits [45,46,47]. In this work, we investigated the impact of marker density on heritability estimates in order to estimate the stable heritability of growth and slaughter traits in meat rabbits (Figure 1). To our knowledge, this is the first research to utilize genome-wide markers to estimate heritability in meat rabbits.

For meat rabbits, there is less information on heritability estimates for growth and slaughter traits. Previously, the heritability for weaning weight in French White rabbits estimated from pedigree information was 0.13 [48], and the heritability for weight at 70 days of age in a crossbred strain of meat rabbits was 0.12 [49]. By exploring the effect of marker density on estimated heritability, we estimated that we could obtain stable heritability for growth and slaughter traits in meat rabbits at a 50 K marker density.

### 4.2. Appropriate Marker Density for Genomic Prediction

In this study, we also investigated the effect of marker density on prediction accuracy to determine the ideal marker density for carrying out genomic selection for growth and slaughter traits in meat rabbits (Figure 2; Table 2).

It is clear that increasing the marker density through genome sequencing did not continuously improve the accuracy of GS [50]. In this study, the marker density played an important role in the improvements in prediction accuracy below 50 K; marker density was positively correlated with GS prediction accuracy in meat rabbit populations within a certain range. Nevertheless, the prediction accuracy exhibited slight fluctuations at marker densities above a threshold of 50 K. A density of 50 K is commonly used in livestock genetics and breeding [51,52,53,54], as also demonstrated in Angora rabbits [55]. Although the baselines for marker density in other species were different, a similar phenomenon was observed in all [56]. Thus, 50 K, a relatively low marker density, was sufficient to produce high prediction accuracy (Figure 2; Table 2).

### 4.3. Different Genome Prediction Models Using Imputation-Based Sequence Data

Genome-wide selection studies center on the accuracy of genomic breeding value estimation. Current genomic selection studies mainly focus on single-trait analyses [57]. However, many traits are genetically correlated with each other, and it has been shown that multi-trait models have higher prediction accuracy than single-trait models [58].

In this work, we observed a significant increase in GEBVs for almost all traits when using the multi-trait model compared to the single-trait model (Figure 3; Table 3). This comparison was performed using an expected dosage-based G matrix based on populated 1.74X sequencing data. However, the benefits of using the multiple-trait model are likely to be applicable to other situations, such as the use of simulated datasets or other animal models, such as the single-step approach, as supported by previous studies [59,60]. In genomic prediction, the multiple-trait model was more accurate than the single-trait model in estimating genomic breeding values for low-heritability traits, particularly when associated with high-heritability traits, as it exploited correlations between traits (Table 1, Table 3 and Table 4). In contrast, the accuracy of the multi-trait model was either similar to or lower than that of the single-trait model for traits with low genetic correlation and low heritability (Table 1, Table 3 and Table 4) [6,59]. The reason for this may be that multi-trait models can utilize information about the correlation of breeding values between different quantitative traits in a population.

The feed conversion ratio is an important growth trait in various livestock and poultry, including meat rabbits, and its genetic improvement is of great importance due to its significant effects on breeding gains, production costs, and overall economic performance [60]. However, the measurement of FCR is more complicated than that of common growth traits, and its genetic improvement is slower. GS for feed conversion ratios in mvLMM can address the problem. This study demonstrated that the use of a multiple-trait model of W49, W70, and FCR can effectively improve the prediction accuracy for FCR. This improvement is likely attributable to the higher genetic correlation between the traits [59]. Therefore, the multi-trait GBLUP model may show better overall accuracy than the single-trait GBLUP model in the prediction of GEBVs for both growth and slaughter traits, especially when there are high genetic correlations between the traits.

To sum up, this study will provide a reliable basis for the practical application of GS in meat rabbit breeding, and we expect it to provide a strategy for the next step in molecular breeding and continuous optimization of breeding programs.

## 5. Conclusions

In this study, we used low-coverage whole-genome sequencing with an average sequencing depth of 1.74× to perform genomic selection for meat rabbits. A total of 20,125,019 high-quality SNPs with an imputation accuracy greater than 98% were produced when this sequencing was combined with the genotype imputation. We randomly selected 0.5 K, 1 K, 3 K, 5 K, 10 K, 50 K, 100 K, 500 K, 1 M, and 2 M markers from the original markers to compare the heritability estimation and genome prediction; we thereby confirmed that 50 K was the optimal marker density. The multi-trait model improved the GEBVs for almost all traits compared to the single-trait model, which is particularly relevant for breeding. In addition, we observed that the improvement in GEBVs in the multi-trait model was almost proportional to the genetic correlation between traits. This is the first reported study to estimate heritability in meat rabbits using genome-wide markers. Therefore, we expect this research to provide strategies for the early GS of meat rabbit traits and the optimization of breeding programs.

## Figures and Tables

**Table 1:** Estimation of heritability based on a single-trait model for original sequence data.

Trait	Heritability
W35	0.176 ± 0.044
W49	0.205 ± 0.044
W70	0.121 ± 0.037
FCR	0.054 ± 0.028
EW	0.216 ± 0.003
HEW	0.205 ± 0.057
KW	0.153 ± 0.051
HLW	0.258 ± 0.063
FLW	0.082 ± 0.048

W35 represents the weight at 35, W49 represents the weight at 49, W70 represents the weight at 70, FCR represents the feed conversion ratio, EW represents the eviscerated weight, HEW represents the half-eviscerated weight, KW represents the kidney weight, HLW represents the hind leg weight, and FLW the represents fore leg weight.

**Table 2:** The accuracies of genomic prediction under different marker densities.

Trait/Accuracy	0.5 K	1 K	3 K	5 K	10 K	50 K	100 K	500 K	1 M	2 M
W35	0.194 ± 0.0097	0.213 ± 0.0096	0.239 ± 0.0112	0.251 ± 0.0094	0.261 ± 0.0092	0.266 ± 0.0099	0.271 ± 0.0094	0.270 ± 0.0093	0.271 ± 0.0090	0.270 ± 0.0087
W49	0.215 ± 0.0083	0.245 ± 0.0081	0.264 ± 0.0093	0.272 ± 0.0087	0.279 ± 0.0085	0.283 ± 0.0089	0.286 ± 0.0084	0.286 ± 0.0069	0.286 ± 0.0068	0.286 ± 0.0065
W70	0.157 ± 0.0091	0.176 ± 0.0084	0.191 ± 0.0125	0.203 ± 0.0105	0.207 ± 0.0104	0.211 ± 0.0116	0.215 ± 0.0113	0.214 ± 0.0102	0.214 ± 0.0099	0.213 ± 0.0100
FCR	0.031 ± 0.0094	0.032 ± 0.0071	0.041 ± 0.0082	0.033 ± 0.0080	0.039 ± 0.0097	0.038 ± 0.0087	0.039 ± 0.0088	0.041 ± 0.0079	0.041 ± 0.0076	0.041 ± 0.0072
EW	0.110 ± 0.0110	0.126 ± 0.0089	0.141 ± 0.0075	0.149 ± 0.0103	0.156 ± 0.0094	0.161 ± 0.0084	0.159 ± 0.0085	0.161 ± 0.0075	0.160 ± 0.0075	0.160 ± 0.0070
HEW	0.113 ± 0.0104	0.137 ± 0.0080	0.140 ± 0.0066	0.146 ± 0.0095	0.154 ± 0.0083	0.159 ± 0.0077	0.157 ± 0.0077	0.158 ± 0.0068	0.158 ± 0.0061	0.157 ± 0.0059
KW	0.068 ± 0.0125	0.077 ± 0.0137	0.085 ± 0.0159	0.092 ± 0.0150	0.103 ± 0.0150	0.106 ± 0.0154	0.109 ± 0.0155	0.104 ± 0.0152	0.105 ± 0.0149	0.105 ± 0.0138
HLW	0.065 ± 0.0043	0.091 ± 0.0066	0.099 ± 0.0061	0.105 ± 0.0052	0.108 ± 0.0054	0.112 ± 0.0057	0.114 ± 0.0054	0.115 ± 0.0052	0.114 ± 0.0051	0.114 ± 0.0049
FLW	0.032 ± 0.0075	0.036 ± 0.0105	0.039 ± 0.0111	0.044 ± 0.0110	0.045 ± 0.0111	0.051 ± 0.0110	0.050 ± 0.0109	0.051 ± 0.0107	0.049 ± 0.0105	0.051 ± 0.0098

W35 represents the weight at 35, W49 represents the weight at 49, W70 represents the weight at 70, FCR represents the feed conversion ratio, EW represents the eviscerated weight, HEW represents the half-eviscerated weight, KW represents the kidney weight, HLW represents the hind leg weight, and FLW represents the fore leg weight.

**Table 3:** Genomic prediction accuracy based on different GBLUP models.

Trait/GEBV	Single-Trait	W35	W49	W70
W49	0.281 ± 0.0082	0.444 ± 0.0087		
W70	0.212 ± 0.0079	0.326 ± 0.0106	0.432 ± 0.0065	
FCR	0.038 ± 0.0082	0.044 ± 0.0087	0.061 ± 0.0093	0.044 ± 0.0086
EW	0.160 ± 0.0084	0.226 ± 0.0082	0.352 ± 0.0098	0.383 ± 0.0102
HEW	0.157 ± 0.0076	0.221 ± 0.0077	0.354 ± 0.0091	0.386 ± 0.0096
KW	0.105 ± 0.0151	0.097 ± 0.0160	0.190 ± 0.0160	0.208 ± 0.0163
HLW	0.114 ± 0.0055	0.160 ± 0.0046	0.271 ± 0.0068	0.293 ± 0.0076
FLW	0.051 ± 0.0112	0.141 ± 0.0107	0.332 ± 0.0105	0.304 ± 0.0106

W49 represents the weight at 49, W70 represents the weight at 70, FCR represents the feed con-version ratio, EW represents the eviscerated weight, HEW represents the half-eviscerated weight, KW represents the kidney weight, HLW represents the hind leg weight, and FLW represents the fore leg weight.

**Table 4:** Genetic correlation among different traits based on the multi-trait GBLUP model.

Trait/GC	W35	W49	W70
W49	0.810 ± 0.0243		
W70	0.617 ± 0.0303	0.881 ± 0.0227	
FCR	0.191 ± 0.0362	−0.246 ± 0.0352	0.081 ± 0.0340
EW	0.201 ± 0.0390	0.756 ± 0.0324	0.899 ± 0.0115
HEW	0.189 ± 0.0390	0.754 ± 0.0324	0.914 ± 0.0104
KW	0.182 ± 0.0471	0.414 ± 0.0443	0.689 ± 0.0371
HLW	0.160 ± 0.0425	0.654 ± 0.0361	0.732 ± 0.0159
FLW	0.226 ± 0.0421	0.794 ± 0.0372	0.870 ± 0.0214

W49 represents the weight at 49, W70 represents the weight at 70, FCR represents the feed con-version ratio, EW represents the eviscerated weight, HEW represents the half-eviscerated weight, KW represents the kidney weight, HLW represents the hind leg weight, and FLW represents the fore leg weight.

## Data Availability

The datasets presented in this article are not readily available because the data are part of an ongoing study. Requests to access the datasets should be directed to lwj17805429586@163.com (Wenjie Li), wangd_18@163.com (D.W.) or xzfan@sdau.edu.cn (X.F.).

## Supplementary Materials

The following supporting information can be downloaded at: https://www.mdpi.com/article/10.3390/genes15040454/s1, Table S1: Sequencing quality statistics; Table S2: The *p*-values of significance tests for the differences between the prediction accuracies under different marker densities; Table S3: The *p*-values of significance tests for the differences between the prediction accuracies by the statistical models.
